# *In situ* quantification of HER2–protein tyrosine kinase 6 (PTK6) protein–protein complexes in paraffin sections from breast cancer tissues

**DOI:** 10.1038/sj.bjc.6605836

**Published:** 2010-08-10

**Authors:** M Aubele, M Spears, N Ludyga, H Braselmann, A Feuchtinger, K J Taylor, K Lindner, G Auer, K Stering, H Höfler, M Schmitt, J M S Bartlett

**Affiliations:** 1 Institute of Pathology, Helmholtz Zentrum München, German Research Center for Environmental Health, D-85764 Neuherberg, Germany; 2 Endocrine Cancer Group and Breakthrough Research Unit, Cancer Research Centre, Western General Hospital, Edinburgh, EH4 2XR, UK; 3 Department of Radiation Cytogenetics, Helmholtz Zentrum München, German Research Center for Environmental Health, D-85764 Neuherberg, Germany; 4 Department of Oncology and Pathology, Karolinska Institute and Hospital, S-17176 Stockholm, Sweden; 5 Institute of Pathology, Technische Universität München, D-81675 Munich, Germany; 6 Clinical Research Unit, Department of Obstetrics and Gynecology, Technische Universität München, D-81675 Munich, Germany

**Keywords:** PTK6 (BRK), HER2, PLA, protein–protein complexes, formalin-fixed paraffin-embedded (FFPE) breast cancer tissue

## Abstract

**Background:**

Protein tyrosine kinase 6 (PTK6; breast tumour kinase) is overexpressed in up to 86% of the invasive breast cancers, and its association with the oncoprotein human epidermal growth factor receptor 2 (HER2) was shown *in vitro* by co-precipitation. Furthermore, expression of PTK6 in tumours is linked with the expression of HER2.

**Method and results:**

In this study, we used the proximity ligation assay (PLA) technique on formalin-fixed paraffin sections from eighty invasive breast carcinoma tissue specimens to locate PTK6–HER2 protein–protein complexes. Proximity ligation assay signals from protein complexes were assessed quantitatively, and expression levels showed a statistically significant association with tumour size (*P*=0.015) and course of the cancer disease (*P*=0.012).

**Conclusion:**

Protein tyrosine kinase 6 forms protein complexes with HER2 in primary breast cancer tissues, which can be visualised by use of the PLA technique. Human epidermal growth factor receptor 2–PTK6 complexes are of prognostic relevance.

Although cytoplasmic protein tyrosine kinase 6 (PTK6; breast tumour kinase (BRK)) is overexpressed in up to 86% of breast carcinomas (Ostrander *et al*, 2007; Harvey *et al*, 2009; Brauer and Tyner, 2010), its physiological role is rather unclear. However, various *in vitro* studies have suggested its possible involvement in modulating signal transduction of human epidermal growth factor receptor (HER) tyrosine kinases (Haegebarth *et al*, 2005; Zhang *et al*, 2005; Ostrander *et al*, 2007; Aubele *et al*, 2008), as well as influencing transcriptional activities (Liu *et al*, 2006; Weaver and Silva, 2007) and proliferation (Harvey and Crompton, 2003). Using immunohistochemistry on breast cancer tissues, PTK6 expression was shown to be associated with the expression of HER receptors (Aubele *et al*, 2007, 2008; Xiang *et al*, 2008), and with the course of the breast cancer disease (Aubele *et al*, 2007).

In this study, we applied the proximity ligation assay (PLA) technique (Söderberg *et al*, 2006), to examine the presence of PTK6–HER2 protein–protein interactions in 80 primary invasive breast carcinoma tissue specimens. As a result, we give evidence that PTK6–HER2 protein complex formation is associated with tumour size and patient outcome.

## Materials and methods

### Transfection of T47D cells

A total of 10^6^ T47D cells growing in antibiotic-free RPMI medium (Aubele *et al*, 2008) were transfected with Lipofectamine 2000 (Invitrogen, Carlsbad, CA, USA) and siRNAs (each 200 pmol, Thermo Scientific, Dharmacon, Chicago, IL, USA) targeting PTK6 and HER2, respectively, according to the manufacturers’ instructions. As controls, we added transfection reagents without siRNAs to the control cells (mock transfection), and performed transfection of the control cells with siRNA targeting GAPDH. After 24 h, one part of the cells was further cultivated in complete medium for 48 h and then used for protein isolation and western blot analysis. The other part of cells was placed on microscopic slides (2.5 × 10^5^ cells per slide; SuperFrost, Carl Roth, Karlsruhe, Germany) and further cultivated for 48 h. Subsequently, the slides were washed in PBS and fixed with 4% paraformaldehyde for 15 min at 22°C.

### Western blot analysis

T47D cells were washed in PBS, sedimented, and lysed as described previously (Aubele *et al*, 2008). Then, PTK6, HER2, and GAPDH protein expressions were detected by primary antibodies against PTK6 (sc-1188, Santa Cruz, Biotech, Heidelberg, Germany), GAPDH (sc-25778, Santa Cruz Biotech, Heidelberg, Germany), HER2 (A0485, DAKO, Glostrup, Denmark), and tubulin (T5168, Sigma, St Louis, MO, USA) as loading control. Peroxidase-conjugated secondary antibodies were added (NA934V and NA931V; GE Healthcare, Chalfont St Giles, Buckinghamshire, UK). Band intensities were quantified densitometrically relative to the appropriate control bands using the Molecular Imager ChemiDoc XRS and the software Quantity One (Bio-Rad Laboratories, Hercules, CA, USA).

### Patients and tumour samples

Tumour samples from 80 patients were randomly selected, and all tumour patients were surgically treated. None of the patients received any preoperative treatment, and there was no correlation between the adjuvant treatment regimes and our results. The median time of follow-up of patients was 139 months (maximum 252 months) with 24 (30%) of the patients experiencing distant disease recurrences. Further information of the samples is summarised in Table 1. For all of the patients, the immunohistochemical data for HER2 status (Hercep test, K5204, DAKO, Hamburg, Germany) and for PTK6 expression (BRK, sc-1188, Santa Cruz, Biotech, Heidelberg, Germany) were available as published elsewhere (Aubele *et al*, 2008).

### Proximity ligation assay technique

From tissue microarray (TMA), blocks containing primary breast cancer tissues of (Aubele *et al*, 2007) 5 μm thick sections were cut, deparaffinised, and subjected to antigen retrieval by microwave cooking in 0.01 M citrate buffer (pH 6.0) at 1000 W for 30 min. From here on the TMA sections and the T47D cells were treated identically. After blocking (purchased by Olink Bioscience, Uppsala, Sweden) the following primary antibodies were used: for PTK6 (BRK, anti-rabbit, sc-1188, Santa Cruz Biotech, Heidelberg, Germany, 1 : 50); for HER2 (Novocastra, anti-mouse, NCL-L-CB11, 1 : 7). The PLA protocol was followed according to the manufacturers’ instructions (Olink Bioscience, Uppsala, Sweden), with incubation of the primary antibodies at 4°C over night. Proximity ligation assay minus and PLA plus probes (containing the secondary antibodies conjugated with oligonucleotides) were added and incubated 2 h at 37°C. Afterwards, further oligonucleotides are added, allowed to hybridise to the PLA probes, and ligase joins the two hybridised oligonucleotides to a closed circle. The DNA is then amplified (rolling circle amplification), and detection of the amplicons was carried out using the ‘563 detection kit’ (including Hoechst 33342 dye nuclear staining), resulting in red fluorescence signals. The sections were mounted with Vectashield mounting media (Vector Lab., Inc., Burlingame, CA, USA).

### Image acquisition and evaluation of signal counts

The hybridised slides were viewed under a confocal laser scanning microscope (AxioImager, Zeiss, Jena, Germany) equipped with the Apotome Extension (Zeiss Vision, Jena, Germany) and appropriate filters (signals: 563/581 nm, nuclei: 352/455 nm excitation/emission). Within each tissue spot optical sections were captured from three to five different tumour areas at a distance of 0.3 μm using a C-Apochromat × 63/1.2 W objective. Three-dimensional image projections were calculated (AxioVision software, Zeiss, Jena, Germany), converted to TIF format, and signals from protein complexes were evaluated using the Definiens Enterprise Image Intelligence Suite software (Definiens, Munich, Germany). A specific rule set was defined to detect and quantify semantic classes (‘signal’ and ‘nucleus’), based on fluorescence layer, intensity, and shape. Finally, for each image the mean number of protein complex signals (mean PLA) was calculated per 1000 pixels of tissue area, and subjected to statistical evaluation.

### Statistics

Correlations among the mean PLA signal counts and the immunohistochemical and histopathological parameters were examined by Spearman's rank correlation test. For univariate survival analysis, Kaplan–Meier curves were calculated for distant metastasis-free survival of patients, and differences between the groups were tested with the log-rank χ^2^ value. Multivariate analysis was carried out using Cox proportional hazards regression and a combined stepwise selection algorithm (SAS Inst., Cary, NC, USA). All parameters reaching a significance level of *P*⩽0.15 in univariate analysis were offered to multivariate analysis. In all, other tests statistical significance was considered proven if *P*⩽0.05.

## Results

### Down-regulation of PTK6 and HER2 proteins in T47D cell line

**Figure 1:**
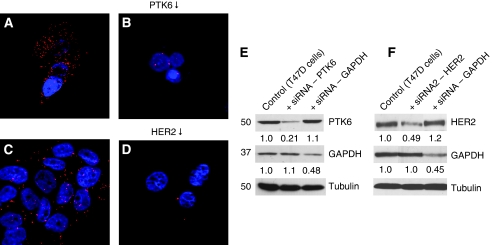
Protein tyrosine kinase 6–HER2 complex formation in T47D cells as visualised by the PLA technique. Control T47D cells (**A**, **C**) and T47D cells after siRNA-mediated down-regulation of PTK6 (**B**) or HER2 (**D**). Western blot analysis of proteins extracted from T47D cells after transfection with siRNA targeting PTK6 (**E**) and HER2 (**F**), respectively; control cells were treated with transfection reagents only or with siRNA targeting GAPDH; (tubulin=loading control).

The need for presence of both, HER2, and PTK6 proteins for detection of PTK6–HER2 protein complexes, was proven by using T47D cells, which slightly overexpress both, HER2 and PTK6. Proximity ligation assay was carried out after down-regulation of PTK6 and HER2, respectively, using specific small interfering RNAs (siRNAs). As shown in Figure 1, cells transfected with siRNA showed significantly reduced signal counts when compared with the non-transfected control cells. These results are corroborated by western blot analysis (Figure 1E, F) showing PTK6 and HER2 down-regulation after siRNA transfection to 21 and 49% of untreated controls, respectively.

### Visualisation and statistical evaluation of PTK6–HER2 protein complexes in tumour tissue

**Figure 2:**
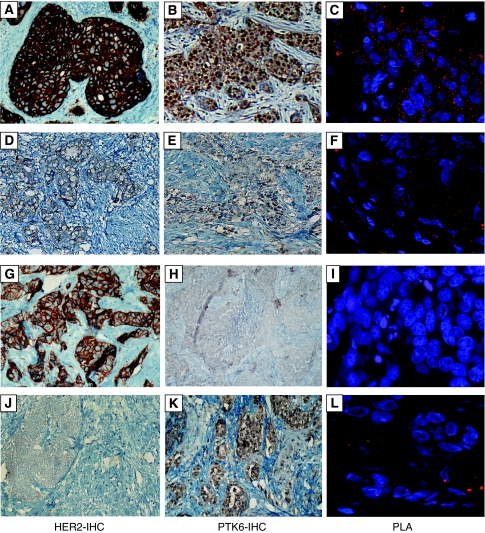
Examples for PLA on tumour tissue sections showing PLA results and the corresponding results obtained by immunohistochemical staining of PTK6 and HER2. Strong staining for HER2 (3+, **A**) and PTK6 (2+, **B**) resulted in high signal counts of protein complexes (**C**); weak positivity for both proteins (HER2: 1+, **D**; PTK6: 1+, **E**) resulted in low signal count (**F**). Further examples are given for HER2 high (**G**) / PTK6 low (**H**) expression and for HER2 low (**J**) / PTK6 high (**K**) expression, both resulting in rare PLA signals (**I**, **L**).

Eighty primary invasive breast carcinomas were analyzed using the PLA technique, using primary antibodies against both HER2 and PTK6 proteins. The mean number of PLA signals detected in all of the cases was compared with the data obtained by immunohistochemistry for PTK6 and HER2, and with the histopathological data and the clinical course of patients. Examples of PLA on tumour tissue specimens are shown in Figure 2 together with corresponding images from PTK6 and HER2 immunohistochemistry.

From the histopathological parameters (tumour size, lymphnode status, histological grade, oestrogen, and progesterone receptor status), only tumour size was significantly correlated (*P*=0.015) with the mean number of PLA signals. The IHC from HER2 (*P*=0.19) and PTK6 (*P*=0.1) were not significantly associated with the PLA signal counts.

**Figure 3:**
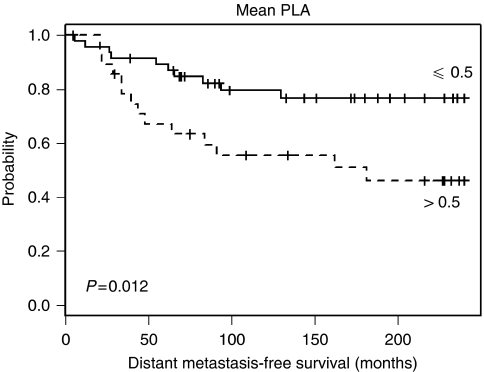
Kaplan–Meier survival curves illustrating probability of distant metastasis-free survival of breast cancer patients according to their HER2–PTK6 protein complex status. Patients with low PLA signals (⩽0.5, solid line) are associated with better prognosis (21% of patients developed distant metastases), whereas from patients with higher PLA signal counts (>0.5, dashed line) 50% suffer from distant metastases. (Mean PLA was defined as mean number of PLA signals per 1000 pixels of tumour area in the section).

With respect to the distant metastasis-free survival (univariate) the mean PLA signals showed a significant univariate association with distant metastasis-free survival of patients. As shown in Figure 3, low signal counts (⩽0.5 per 1000 pixels of tumour area, *n*=48 patients) were associated with better prognosis with 20.8% of patients with distant metastasis, whereas from the patients with >0.5 signals (*n*=28) 50% suffer from metastasis (*P*=0.012). Furthermore, IHC-PTK6 (*P*=0.018, inverse), tumour size (*P*=0.01), and lymph node status (*P*=0.004) were also significant in univariate analysis, whereas no significance level was reached by IHC-HER2 (*P*=0.35), ER (*P*=0.9), and PrR (*P*=0.36).

Although the number of tumours is very small, we carried out multivariate analysis offering all parameters significant in univariate analysis: the prognostic value of the lymphnode status (*P*=0.008) was further improved by IHC-PTK6 (*P*=0.006) and by the mean PLA signals (*P*=0.01).

## Discussion

The function of PTK6 remains poorly understood. Protein tyrosine kinase 6 has been linked to cell differentiation, growth regulation, and apoptosis (Harvey and Crompton, 2003; Brauer and Tyner, 2010), and there is increasing evidence that it interacts with HER receptors (Kamalati *et al*, 1996, 2000; Chen *et al*, 2004; Haegebarth *et al*, 2005; Aubele *et al*, 2008; Xiang *et al*, 2008) and downstream signalling molecules (Zhang *et al*, 2005; Ostrander *et al*, 2007; Aubele *et al*, 2008). So far, PTK6 interacting proteins were identified in *in vitro* studies using immunoprecipitation and western blot analysis. In breast cancer tissue significant correlation was identified between the expression levels of PTK6 and HER2 (Born *et al*, 2005; Aubele *et al*, 2007, 2008; Xiang *et al*, 2008). Thus, potentially, PTK6 may represent a therapeutic target in clinical management of breast cancer in addition to the established anti-HER2 therapy with the humanised antibody trastuzumab, which targets HER2, but progression is common within 1 year (Nahta and Esteva, 2007).

The PLA technique enables accurate visualisation, subcellular localisation, and quantitative evaluation of protein–protein interactions, as, for example, was shown for complexes of prostate specific antigen with α1-protease inhibitor (Zhu *et al*, 2009), and for EGF receptor–HER2 complex formation *in vitro* (Leuchowius *et al*, 2009).

In this study, we used the PLA technique to show for the first time ever that PTK6 forms protein complexes with HER2 in paraffin tissues from invasive breast carcinomas. Our results on tumour tissues are corroborated by down-regulation of each of these proteins in T47D cell line, resulting in reduced PLA signal counts. In tumour tissues, we identified significant correlation of PLA signals with tumour size and with distant metastasis-free survival of patients. In the context of our previous findings, in which PTK6 expression was shown to be linked to a better prognosis, the correlation of PTK6–HER2 protein complexes with occurrence of metastasis seems to be inconsistent. However, the associations of HER2 with bad prognosis (*P*=0.35) and of PTK6 with good prognosis (*P*=0.018, inverse correlation) were confirmed in this study. The association of the mean PLA signals with bad prognosis may be explained by the fact that PLA signals do not necessarily reflect the expression of the single proteins, but represent a feature independent from the single protein expressions.

Our findings show that HER2–PTK6 protein complexes are of prognostic value and that PTK6 may be considered as additional breast cancer biomarker and potential target for anticancer therapy.

## Figures and Tables

**Table 1:** Histopathological parameters of the tumours

**Parameter**	**Number of patients**
Lymphnode status 0/+	51, 29
	
*Tumour size*	
<2 cm, ⩾2<5 cm, ⩾5 cm	58, 20, 2
	
*Histological grade* a	
(1, 2, 3)	13, 42, 25
	
*Histological type*	
Invasive ductal	67
Lobular	7
Else	6
	
*Estrogen receptor*	
Negative, low, high	20, 27, 33
	
*Progesterone receptor*	
Negative, low, high	38, 17, 25
	
*IHC-HER2*	
0/1+	56
2+/3+	24
	
*IHC-PTK6*	
0/1+	28
2+/3+	52

Abbreviations: HER2=human epidermal growth factor receptor 2; IHE=immuno histochemistry; PTK6=protein tyrosine kinase 6.

a Grading according to Elston and Ellis (1991).
